# Anxiety and Depression in Children with Irritable Bowel Syndrome—A Narrative Review

**DOI:** 10.3390/diagnostics15040433

**Published:** 2025-02-11

**Authors:** Daniela Pop, Sorin Claudiu Man, Dorin Farcău

**Affiliations:** 1 Third Pediatric Discipline, “Iuliu Hațieganu” University of Medicine and Pharmacy, 400217 Cluj-Napoca, Romania; 2 Third Pediatric Department, Emergency Hospital for Children, 400217 Cluj-Napoca, Romania; 3 Nursing Discipline, “Iuliu Hațieganu” University of Medicine and Pharmacy, 400217 Cluj-Napoca, Romania

**Keywords:** irritable bowel syndrome, anxiety, depression, children

## Abstract

Irritable bowel syndrome (IBS) is one of the most common functional gastrointestinal disorders diagnosed in children. It has a complex pathophysiology with several potential risk factors, including psychological disorders like anxiety and depression. This paper aimed to find genetic, pathophysiological, and clinical links between psychological factors (mainly anxiety and depression) and IBS in children. Impairment of the gut–brain communication and signalling of serotonin is responsible for both gastrointestinal and psychological disorders. Childhood psychological events seem to be linked to gastrointestinal symptoms not only in childhood but also in adulthood. Evidence of the efficacy of therapies targeting psychological disorders (antidepressant, hypnotherapy, and cognitive behavioural therapy) in children with IBS was evaluated. Further studies that use updated criteria for IBS and uniform questionnaires and outcome measures are needed to draw reliable conclusions regarding the connection between psychological factors and IBS.

## 1. Introduction

Irritable bowel syndrome (IBS) is one of the most common functional gastrointestinal disorders diagnosed in children, with a significant impact on daily activities. Symptoms of IBS are bothersome for children and worrying for parents, leading to repeated referrals to the pediatrician or pediatric gastroenterologist in an attempt to find a cause that can be quickly eliminated so that the child does not miss school, and so the parent will not be late to or miss work. Diagnosing this disease implies the absence of an organic, biochemical, inflammatory cause, a conclusion which will likely displease the parent. Discontent is often accentuated by doctors’ suggestion that a psychologist should be involved in the child’s evaluation to find possible social and psychological distress factors that might be linked to the digestive symptoms.

The prevalence of IBS in children varies in different parts of the world, from 2.8% in the United States to 22.9% in Turkey [1]. Currently, IBS is diagnosed in children based on the Rome IV criteria released in 2016. IBS is diagnosed in a child older than 4 years of age presenting with at least 2 months of abdominal pain associated with changes in intestinal transit, in which appropriate medical evaluation did not find a cause for the symptoms [2]. There are four subtypes of IBS described in children: constipation-predominant, diarrhea-predominant, mixed or alternating constipation and diarrhea, and an unclassified type [1]. The prevalent subtype of IBS in children is IBS manifested with constipation (45%), with a significantly higher frequency in girls [3]. IBS with a predominance of diarrhea is more frequent in boys [3].

Research in pediatric neurogastroenterology has aimed to find the underlying cause of IBS. IBS has a complex pathophysiology, including impairment of the gut microbiota, motility disorders, visceral hypersensitivity, disturbances of intestinal permeability, and altered immune activation [1]. The following various perinatal or childhood risk factors seem to have varying influence on triggering these mechanisms: sex, low birth weight, birth through caesarean section, gastric suction at birth, short duration of breastfeeding, young maternal age, family history of IBS, parents with mental illness or substance abuse, parental deprivation or over-interference, gastrointestinal infection, psychological factors, sexual abuse, socioeconomic and environmental factors, child abuse, pets, air pollution, antibiotic use, diet, poor sleep, allergic diseases, etc. [4].

It is known that anxiety and depression are risk factors for functional abdominal pain-related disorders in children. The severity of symptoms is higher in children with abdominal pain-related disorders with associated psychological risk factors. This narrative review synthesizes the genetic, pathophysiological, clinical, and treatment-related connections between psychological risk factors and IBS in children.

## 2. Aim

The question that started this research was what is the current evidence concerning the links between anxiety and depression and IBS in children? This review aims to provide an image of what is known about the link between IBS and psychological factors (mainly anxiety and depression) in children diagnosed with this functional gastrointestinal disorder. Genetic, pathophysiological, and clinical links, and responses to common treatment options, are described. The focus is mainly on children with IBS but, especially where data are lacking in this category of patients, reference to adult studies was made as well. This research focuses on articles in English published on PubMed, Scopus, Google Scholar, Web of Science, and Cochrane Library before September 2024, using keywords like ”irritable bowel syndrome”, ”children”, ”anxiety”, ”depression”, tricyclic antidepressants”, “amitriptyline”, “cognitive behavioural therapy”, and “hypnotherapy”. Original articles, meta-analyses, and systematic reviews are included in the analysis.

## 3. Genetic Links Between IBS and Psychological Disorders

Bonfiglio et al., in a study published in 2018, identified a genome-wide significant association (variant rs10512344) on chromosome 9q31.2 in women with IBS [5]. Eijsbouts et al. brought genetic research a step further, showing a genome-wide overlap for IBS and psychological disorders, especially for anxiety, depression, neuroticism, and schizophrenia [6]. Four genes were found to influence mood and anxiety disorders in patients with IBS: *NCAM1*, *CADM2*, *PHF2*, and *DOCK9*. *NCAM1*, *PHF2*, and *DOCK9* are also expressed in the enteral nervous system [6].

Ng QX et al. suggest that post-traumatic stress disorder leads to epigenetic changes in glucocorticoid gene expression and neuroendocrine pathways [7]. The consequence is inflammation and impaired function of the hypothalamic–pituitary–adrenal axis and serotonin [7].

## 4. Pathophysiology Links Between IBS and Psychological Disorders

The gut–brain interaction involves the central, autonomic, and enteral nervous systems, as well as the neuro-endocrine and neuro-immune systems [1]. The neuroendocrine stress response is controlled by the paraventricular hypothalamic nucleus through the hypothalamic–pituitary–adrenal axis [8]. Stress induces an increase in corticotropin-releasing factor. Corticotropin-releasing hormone increases colonic motility and pain in adult patients with IBS [8].

A possible impairment of gut–brain communication, mediated by the hypothalamic–pituitary–adrenal axis and the autonomic nervous system in functional gastrointestinal disorders associated with abdominal pain, such as IBS, has been studied in children [9]. Gulewitsch et al.’s study showed that children with functional abdominal pain disorders seem to have a downregulated hypothalamic–pituitary–adrenal axis capacity, probably as a consequence of chronic stress [9]. There was no evidence in that study for parasympathetic withdrawal during stress [9].

In their study, Sjolund et al. support the idea of a bidirectional gut–brain interaction that leads to symptoms of IBS and other abdominal pain-related functional gastrointestinal disorders [10]. Abnormalities in the levels and signalling of serotonin (5-hydroxytryptamine-5-HT), an essential neurotransmitter in the enteral nervous system, lead to both intestinal and extraintestinal symptoms, including diarrhea, constipation, bloating, nausea, vomiting, altered pain perception, anxiety, obsessive-compulsive disorders, depression, and phobias [1,11].

Studies in adult patients with IBS show that visceral hypersensitivity may play a role in the pathophysiological mechanisms of this disease [12,13,14]. Elsenbruch et al. aimed to describe the role of anxiety and depression in pain processing in adult patients with IBS [12]. They found that anxiety and depression alter the perception of pain [12]. Dorn et al. document an increased tendency to report pain in adults with IBS-associated anxiety and depression [13]. In post-infectious IBS adult patients, Sundin et al. report that a reduced fecal microbiota in both the mucosa and intestinal lumen was found in patients with anxiety and/or depression [14].

Brain imaging studies like MRI and functional MRI have shown structural changes in the hypothalamus, grey matter density, volume, and cortical thickness in regions involved in emotional modulation or pain modulation in patients with IBS [15].

Pediatric patients with IBS have lower cortical thinning in the dorsomedial and dorsolateral prefrontal cortex and posterior parietal cortex, and they show cortical thickening in the posterior cingulate cortex [16]. A study was conducted by Hubbard et al. in 19 adolescents (mean age ± SD = 16.00 ± 2.09 years) diagnosed with IBS based on Rome III criteria, compared with a healthy control group [16]. In these patients, functional disability was positively correlated with total anxiety scores [16]. Cortical thickening in the left posterior parietal cortex, supramarginal gyrus, the right inferior temporal, and fusiform gyri was associated with greater anxiety scores [16]. The authors also noted a strong negative correlation between cortical thickness in the right dorsolateral prefrontal cortex and pain catastrophizing [16]. It is undetermined whether these structural and functional brain changes precede or follow the symptoms of IBS [16]. The authors conclude that these brain changes may have long-term consequences on psychosocial functioning, anxiety and depression [16].

Bhatt et al. aimed to find structural differences in the grey matter volume in the brain, and its associated resting-state abnormalities in functional connectivity, in girls with IBS, compared to healthy controls [17]. They also wanted to correlate these changes with anxiety levels, pain-related anxiety, and pain levels [17]. The study included 32 girls aged between 7 and 17 years diagnosed with IBS based on Rome III criteria [17]. The participants of the study with IBS had a lower grey matter volume in the prefrontal cortex, basal ganglia, and anterior mid-cingulate, altered functional connectivity between multiple brain networks, and showed altered relationships between pain sensitivity and brain structure [17].

A recent review by Mayer et al. gathered growing evidence from both clinical and preclinical studies in support of a common genetic, structural, and functional background for gastrointestinal and psychological disorders [18]. In addition to these shared factors, environmental influences, especially during childhood, play an important role [18].

## 5. Anxiety and Depression in Adults with IBS

Correlations between anxiety and depression and different subtypes of IBS; connections between psychological factors and other symptoms of IBS besides abdominal pain or changes in bowel pattern, like bloating; and the influences of psychological factors in different ethnic groups, and dietary and cultural influences, are not well studied in children with IBS.

In adult patients, however, a more recent meta-analysis by Hu et al. explored the prevalence of anxiety and depression in adult patients with different IBS subtypes and in healthy controls [19]. IBS with mixed symptoms, both diarrhea and constipation, was associated with the highest levels of anxiety and depression, followed by IBS manifested as constipation and IBS manifested as diarrhea [19]. Patients with IBS manifested predominantly by constipation had the highest prevalence of anxiety and depression (40% and 38%, respectively) [19]. In a systematic review and meta-analysis that aimed to compare depression and anxiety levels in adult patients with IBS of different subtypes, both depression and anxiety levels were found to be higher in adult patients with IBS compared with healthy controls [20]. This difference was also noted in the subgroup analysis of IBS subtypes [20]. The translation of English questionnaires into different languages and cross-cultural validation would improve global epidemiological studies in both IBS and psychological disorders [20].

One of the most bothersome symptoms in patients with IBS is bloating. Hod et al. found a significant correlation between bloating severity and both depression and somatization in a study of adult patients with IBS [21]. Anxiety scores were higher in IBS patients with bloating as compared with IBS patients without boating, but these differences did not reach statistical significance [21]. Ryu et al. also report an association between depression and bloating [22]. The question remains as to the association of abdominal pain and psychological symptoms, and whether the gastrointestinal symptoms precede or follow the psychological ones [22].

## 6. Childhood Psychological Events with Impact on Adult IBS

About 50% of adult patients with IBS report mental symptoms like anxiety or depression [23]. Illness anxiety is considered a risk factor for the development of IBS in adults [24]. A question that has not been answered is whether symptoms like anxiety or depression trigger gastrointestinal symptoms or abdominal pain, and whether changes in stool patterns lead to psychological distress. Answering this question would optimize treatment options in patients with IBS.

Studies performed in adult patients with IBS reveal a strong link between events with psychological impact, taking place in childhood or later in life, and the development of gastrointestinal symptoms [25,26,27]. In a systematic review, Chitkara et al. state that symptoms related to functional gastrointestinal disorders experienced in childhood are likely to persist into adulthood [28]. Factors that may contribute to this situation are socioeconomic status, traumatic events during infancy or childhood, such as physical, emotional, or sexual abuse, and parental separation or divorce. Park et al., in a study performed on adults with IBS, found that early life events like incarceration or mental illness in a member of the household and emotional abuse are significant predictors of IBS [29]. IBS is also linked to post-traumatic distress syndrome in adult patients [7]. Berens et al.’s study suggest that adverse childhood experiences and illness anxiety are significantly increased in patients with IBS, with significant correlations for women [30].

Howell et al. conclude in their study that chronic abdominal pain in children progresses into adulthood gastrointestinal disorders, particularly in children aged between 7 and 9 years [31]. However, emotional distress was not correlated with this finding [31].

Anxiety and depression are more likely to be associated in children diagnosed with IBS who have a family history of this disease [32]. A study from a group with significant research experience in clustering functional gastrointestinal disorders within families [33] found a positive correlation between mothers and children for somatization. Multiple independent factors and behaviours are transmitted from one generation to another, influencing a child’s response to somatic sensations [33]. Ramchandani et al. showed that in children aged between 2 and 6 years, recurrent abdominal pain was associated with higher scores for anxiety and depressive disorders in the mothers [34].

Campo et al. showed that anxiety disorders seem to be diagnosed in adults who had functional abdominal pain associated with anxiety in childhood [35]. Later, a study from the same group found that an anxiety disorder was diagnosed in almost 80% of children with recurrent abdominal pain, with separation anxiety being the most frequent [36]. Almost 43% of children exhibited a depression disorder [36].

## 7. Functional Gastrointestinal and Psychological Disorders in Children

Zia et al., in a systematic review, found that anxiety and depression are associated with abdominal pain-related disorders of the gut–brain interaction in children [37]. Anxiety and depression were associated with a two-fold increased risk of abdominal pain-related disorders of the gut–brain interaction [37]. Also, these two psychological factors are associated with the persistence and chronicity of abdominal pain-related disorders of the gut–brain interaction [37].

Waters et al. found that the incidence of functional gastrointestinal disorders was significantly higher in children with anxiety disorders in comparison with non-anxious children (40% versus 6%) [38]. The severity of symptoms was also higher [38]. Yacob et al. report a prevalence of 51.5% of anxiety or depression in children with pain-predominant functional gastrointestinal disorders (including IBS), as compared with controls (8.8%) [39].

Most of the studies in children focus on finding a relationship between psychological factors, like anxiety and depression, and functional gastrointestinal disorders manifested as abdominal pain, in general, and not specifically IBS. However, Rutten et al. found no differences in anxiety and depression scores in children with IBS compared with children with functional abdominal pain syndrome [40]. Further, 28% of children with IBS were greatly anxious, and 34.8% had depression [40]. The authors support the idea that IBS and functional abdominal pain syndrome are one underlying functional disorder with different expressions if psychological characteristics are considered [40]. The overlap in symptoms of gastroesophageal reflux disease, functional dyspepsia, and IBS is also noted by Colombo et al., who find that children with IBS or functional dyspepsia who report heartburn have increased anxiety, depression, and sleep disturbances [41].

A study regarding chronic pain in children reported that 55% of the children had more than one pain diagnosis simultaneously [42]. Chronic abdominal pain-related diagnoses were found in 22.3% of the children enrolled in the study. Further, 31.6% of the children with chronic abdominal pain had significant scores for anxiety, and this was 26.7% for depression [42]. Machnes-Maayan et al. found that of 19 children with recurrent abdominal pain (medium age 12.8 ± 3.267 years), 52.6% had psychiatric comorbidities such as different types of anxiety or depressive disorders, obsessive-compulsive, post-traumatic stress, or attention deficit hyperactivity disorders, or phobias [43]. However, the prevalence of associated depressive disorders was lower than in other studies (5.3%) [43].

A study conducted by Shelby et al. prospectively followed children with functional abdominal pain into adolescence and young adulthood to investigate if these patients have a higher risk of anxiety and depressive disorders compared to children without these associations [44]. IBS was diagnosed in 27.4% of the children included in the study. In this study, 51.2% of patients had at least one lifetime anxiety disorder [44]. Social anxiety was the most common disorder, diagnosed in 25.9% of patients with functional abdominal pain [44]. Depressive disorders were diagnosed in 40.1% of participants in the functional abdominal pain group [44]. In a significant number of patients, anxiety disorders continued into adolescence and adulthood [44].

Saps et al. found a seasonal link between functional abdominal pain disorders and anxiety and depression [45]. Consultation rates for abdominal pain decreased by 20-to-25% in the summer months [45]. The same trend was noted for consultations for anxiety and depression (a decrease of 5-to-20% in the consultation rates) [45]. A valid explanation was not found, but the authors speculate that school-related stress could be a cause [45].

## 8. Anxiety and Depression in Children with IBS

A case–control study that included patients with both IBS and recurrent abdominal pain found lower thresholds for visceral perception in patients with IBS compared to controls [46]. Higher scores for anxiety were found in 45% of the total number of patients [46].

Yamamoto et al. evaluated the symptoms and associated factors in a large number of children diagnosed with IBS [47]. Depression or anxiety were strongly associated with IBS, especially in correlation with constipation [48].

Dong et al. prospectively studied risk factors in children diagnosed with IBS [48]. The authors found that, among other factors, anxiety and depression are significant risk factors for IBS in children [48].

In a more recently published prospective study, Hollier et al. tried to see if pain catastrophizing and somatization mediate the relationship between pain severity and anxiety or depression [49]. The authors analyzed children diagnosed with IBS [49]. The study suggests that the association between psychological factors like anxiety and depression and pain severity in children with IBS is mediated by somatization and pain catastrophizing [49]. These two factors might be better treatment targets than anxiety and depression [49].

A retrospective study published by Fu et al. enrolled children fulfilling Rome IV criteria for IBS [32]. The study focused on family history of IBS, a factor which increased the risk of a child developing anxiety and depression and having psychological counselling and antidepressant treatment [32].

Studies that analyzed anxiety and depression in children with IBS are summarized in Table 1.

## 9. Treatment Options Targeting Psychological Factors in Children with IBS

Amitriptyline is a tricyclic antidepressant. Two randomized placebo-controlled trials looked into the effect of amitriptyline in children with IBS. Bahar et al. performed a study in 33 adolescents diagnosed with IBS based on Rome II criteria [50]. In detail, 17 patients (mean age 14.2 years) received placebo, and 16 children (mean age 15.2 years) received amitriptyline [50]. The study found a significant improvement in patients treated with amitriptyline for abdominal pain (periumbilical after 10 weeks and right-lower quadrant after 6, 10, and 13 weeks of treatment) and diarrhea [50]. The authors mentioned parents’ reluctance to participate in the study because of the possibility of their children receiving antidepressant therapy for IBS [50]. Saps et al. aimed to find the efficacy of amitriptyline in children with functional abdominal pain-related disorders (IBS, functional abdominal pain, and functional dyspepsia) [51]. The study included 90 children (mean age 12.7 years) diagnosed with one of these three disorders [51]. The study was completed by 83 patients (43 in the amitriptyline group and 40 in the placebo group) [51]. There was a significant improvement in both groups for depression and somatization, but without significant differences between the two groups [51]. There was a significant improvement in anxiety for children receiving amitriptyline but not for the children receiving placebo [51]. There was a significant decrease in pain but without differences between the two groups of patients [51]. A retrospective study was published by Teitelbaum et al. on children with functional abdominal pain disorders treated with tricyclic antidepressants and included 55 children with IBS; 35 were treated with amitriptyline and 20 with imipramine [52]. Overall, 76.5% of the patients with IBS, including those who showed adverse effects from the medication, responded to the antidepressant medication, with an average duration of response of 12.9 months [52]. A systematic review by de Bruijn et al. concluded that there is insufficient evidence to support the beneficial effect of amitriptyline in children with functional abdominal pain-related disorders [53]. In a more recent study by Seetharaman et al. including 149 children (mean age 11.3 ± 3.5 years) with functional abdominal pain disorders, 75 were treated with amitriptyline and 74 received placebo); there was a significant improvement in the scores for the intensity, frequency, and duration of pain in the children treated with amitriptyline [54]. Of the children who had an extended treatment duration (around 6 months), 89% showed pain improvement [54].

There are no studies on serotonin-selective reuptake inhibitors in children with IBS who also show depression.

There are a number of studies evaluating the effect of alternative treatments like hypnotherapy [55,56], meditation [57], and cognitive behavioural therapy [58] on symptoms related to functional gastrointestinal disorders, including IBS, which offer indirect proof of the association between psychological symptoms and IBS symptoms, as these treatment options are often used in anxiety- and depression-related disorders as well.

A review published in 2023 by Chakraborty et al. found four papers focusing on treatment for cognitive behavioural therapy in functional gastrointestinal disorders: two randomized control trials, one meta-analysis, and one prospective longitudinal study [58]. One of these studies was a randomized controlled trial that included 101 adolescents aged 13–17 years diagnosed with IBS based on Rome III criteria, aiming to investigate the efficacy of internet-based cognitive behavioural therapy in these patients [59]. Here, 47 patients were included in the internet-based cognitive behaviour group and 54 in the wait-list control group [59]. The results of the study showed significant improvement in anxiety, fear, and worry, and quality of life scores, but also a decrease in pain frequency in adolescents with IBS after completing a 10 week programme of internet-based cognitive behavioural therapy [59].

A randomized controlled trial in 126 children with IBS (mean age 13.3 years) was conducted by Rutten et al. [40]. They compared the efficacy of home-based hypnotherapy using a CD with hypnotherapy performed by a therapist [40]. In both groups, anxiety, depression, pain, and somatization scores were improved [40]. Vasant et al. showed that gut-focused hypnotherapy improved both gastrointestinal symptoms scores and anxiety and depression scores [56]. This study included 32 patients with IBS (mean age 15.7 years) [55].

In a retrospective study by He et al., the authors explored a high-quality nursing intervention on the symptoms and negative emotions (among other features) of children with IBS manifested as diarrhea [60]. The study included 60 children aged 6 to 11 years of age [60]. The high-quality nursing included lifestyle and dietary changes, cognitive and psychological intervention, and meditation guidance [60]. Anxiety and depression were evaluated by self-rating scales [60]. The scores for both anxiety and depression were improved after the high-quality nursing intervention [60]. Clinical symptoms of and treatment outcomes for diarrheal IBS were also improved [60]. A recent guideline recommends the assessment of psychological comorbidities in children with IBS [61].

The gut microbiota influences, among other neurotransmitters, the release of serotonin from the enteroendocrine cells, which activate receptors on vagal afferent neurons, the path through which the central nervous system and the enteral nervous system interact [62]. By restoring the microbiome balance, probiotics and prebiotics could positively influence mood, cognitive functions, and stress levels [62]. Diet’s influence on gut microbiome is under extensive study, including in children with IBS [63]. However, the benefit on psychological disorders associated with IBS remains to be studied.

There are few good-quality studies that explore psychological factors in children with IBS. Most of the studies include children with functional gastrointestinal disorders manifested with abdominal pain, and not specifically IBS. Enrolment of a significant number of patients takes years, and follow-up is often difficult. Some of the studies that have been published included a small number of patients and relied on parents’ interpretation of children’s symptoms, or the recollection of past events from childhood in adults with IBS. The questionnaires that have been used to assess psychological factors are different, and the outcome measures used also vary among the studies we found. Most studies focus on the relationship between abdominal pain and psychological factors and do not specify the connections between other symptoms of IBS and psychological factors.

## 10. Conclusions and Future Research

Anxiety and depression are commonly found in children with IBS. Childhood psychological events seem to be linked to gastrointestinal symptoms in childhood and adulthood. Antidepressants, cognitive behavioural therapy, and hypnotherapy improve both gastrointestinal and psychological symptoms in children with IBS. More studies are needed in children to better describe the relationship between anxiety, depression, and other psychological factors; what mediates and influences this relationship; and how treatment options can be tailored to the specific psychological profile of a child with IBS in order to have the best outcome and avoid any persistence of both psychological and gastrointestinal disorders into adulthood. Future longitudinal, well-designed studies should address cultural and dietary differences among different ethnic groups, environmental and social factors, and their influences on the symptoms of children with IBS.

## Figures and Tables

**Table 1:** Studies evaluating anxiety and depression in children with IBS.

Authors	Year of Publication	Study Type	IBS Criteria	Age of Children with IBS	Number of Children with IBS	Results for Associated Psychological Factors
Di Lorenzo et al. [46]	2001	Case–control	Apley criteria for recurrent abdominal pain	mean age 13.0 ± 0.9 years	10	Higher scores for depression and anxiety correlated with duration of symptoms
Dong et al. [48]	2005	Prospective, data collection	Rome II	age range 6 to 18 years	716	Odds ratio was 1.07 for anxiety and 1.13 for depression
Yamamoto et al. [47]	2015	Cross-sectional, population based	Rome III	age range 12–17 years	17,882	Anxiety or depression were present in 10,271 of the patients;odds ratio 1.62
Hollier et al. [49]	2019	Cross-sectional	Rome III	mean age 10.4 years	261	Mediation analysis for anxiety and depression for pain catastrophising and somatization
Fu et al. [32]	2021	Retrospective	Rome IV	mean age 12.4 years	251	Anxiety was found in 21% and depression in 14% of the children with IBS

## Data Availability

Not applicable.
